# Endometrial Cancer-Adjacent Tissues Express Higher Levels of Cancer-Promoting Genes than the Matched Tumors

**DOI:** 10.3390/genes13091611

**Published:** 2022-09-08

**Authors:** Mariusz Kulinczak, Maria Sromek, Grzegorz Panek, Klara Zakrzewska, Renata Lotocka, Lukasz Michal Szafron, Magdalena Chechlinska, Jan Konrad Siwicki

**Affiliations:** 1 Department of Cancer Biology, Maria Sklodowska-Curie National Research Institute of Oncology, 02-781 Warsaw, Poland; 2 Department of Gynecologic Oncology and Obstetrics, Centre of Postgraduate Medical Education, 00-416 Warsaw, Poland; 3 Department of Pathology, Maria Sklodowska-Curie National Research Institute of Oncology, 02-781 Warsaw, Poland; 4 Cancer Molecular and Genetic Diagnostics Laboratory, Maria Sklodowska-Curie National Research Institute of Oncology, 02-781 Warsaw, Poland

**Keywords:** endometrial carcinoma, tumor-adjacent tissues, cancer-promoting genes, field cancerization

## Abstract

Molecular alterations in tumor-adjacent tissues have recently been recognized in some types of cancer. This phenomenon has not been studied in endometrial cancer. We aimed to analyze the expression of genes associated with cancer progression and metabolism in primary endometrial cancer samples and the matched tumor-adjacent tissues and in the samples of endometria from cancer-free patients with uterine leiomyomas. Paired samples of tumor-adjacent tissues and primary tumors from 49 patients with endometrial cancer (EC), samples of endometrium from 25 patients with leiomyomas of the uterus, and 4 endometrial cancer cell lines were examined by the RT-qPCR, for *MYC*, *NR5A2*, *CXCR2*, *HMGA2*, *LIN28A*, *OCT4A*, *OCT4B*, *OCT4B1*, *TWIST1*, *STK11*, *SNAI1*, and miR-205-5p expression. The expression levels of *MYC*, *NR5A2*, *SNAI1*, *TWIST1*, and *STK11* were significantly higher in tumor-adjacent tissues than in the matched EC samples, and this difference was not influenced by the content of cancer cells in cancer-adjacent tissues. The expression of *MYC*, *NR5A2*, and *SNAI1* was also higher in EC-adjacent tissues than in samples from cancer-free patients. In addition, the expression of *MYC* and *CXCR2* in the tumor related to non-endometrioid adenocarcinoma and reduced the risk of recurrence, respectively, and higher *NR5A2* expression in tumor-adjacent tissue increased the risk of death. In conclusion, tissues proximal to EC present higher levels of some cancer-promoting genes than the matched tumors. Malignant tumor-adjacent tissues carry a diagnostic potential and emerge as new promising target of anticancer therapy.

## 1. Introduction

Tissues adjacent to a primary malignant tumor (TA) have been shown to present substantial molecular alterations, such as loss of heterozygosity, aneuploidy, mutations, transcriptomic and epigenetic alterations, protein expression changes, and metabolic disturbances [1,2,3]. Moreover, unique profiles of mRNA and miRNA transcriptomes have been identified in tumor-surrounding tissues in a number of cancer types, including prostate [4], colon [5], and breast carcinomas [6].

As shown in breast and lung cancers, molecular changes in TA may relate to cancer subtype [7,8]. Molecular signatures of the extratumoral microenvironment predicted clinical outcome, e.g., in head and neck, breast, and hepatocellular cancers [9,10,11]. Noteworthy, transcriptional profiles of TA samples were more informative on patient survival than the profiles of the paired tumor samples [12,13]. Hence, tumor-surrounding tissues emerge as an interesting research subject, not only in terms of basic pathogenic mechanisms but also as an important player of tumor progression, carrying a clinical potential. In endometrial cancer (EC), data on molecular changes in tissues neighboring primary tumor are scarce.

We aimed to molecularly characterize tumor-adjacent tissues in patients with EC. Therefore, we analyzed the expression of genes associated with cancer progression and metabolism, *TWIST1*, *SNAI1*, *NR5A2*, *MYC*, *CXCR2*, *STK11*, *POU5F1* (*OCT4*, isoforms *A*, *B*, and *B1*), *HMGA2*, as well as miR-205-5p, in the matched samples of tumor-adjacent tissues and tumors from patients with endometrial cancer, and in the samples of endometrium from patients with uterine leiomyoma.

A large body of evidence indicates multiple oncogenic roles of *MYC* in carcinogenesis, including the pathogenesis of endometrial cancer [14,15,16].

The orphan nuclear receptor, NR5A2, has been implicated in a variety of biological processes, including cholesterol metabolism, steroidogenesis, embryogenesis, inflammation, and stem cell pluripotency [17]. *NR5A2* overexpression and its oncogenic role has been implicated in multiple cancers [18]. *NR5A2* is expressed in endometrial cancer cell lines [19], but its role in EC pathogenesis has not been revealed.

Overexpression of *SNAI1* and *TWIST1*, encoding key regulators of epithelial-to-mesenchymal transition (EMT), significantly contributes to cancer development [20] and has been directly related to chemoresistance [21] and poor prognosis in several cancer types [22]. Previous studies on SNAI1 and/or TWIST1 in EC mostly relied on their immunohistochemical evaluation in tumor cells in the context of clinical variables, and increased SNAI1 and TWIST1 expression has been linked to EC progression or poor prognosis [23,24,25,26].

*STK11* (also known as *LKB1*) has been identified as a tumor suppressor in multiple cancers [27], but in EC, clinical samples have not been evaluated in this respect.

Aberrant expression/signaling of *CXCR2* and of its multiple ligands, including several CXC chemokines GRO-α, plays crucial role in carcinogenesis by promoting cancer cell proliferation, migration, and invasion, as well as angiogenesis and by contributing to chemo- and radioresistance in many cancers [28,29,30,31,32,33,34,35]. Increased *CXCR2* expression has been linked to poor prognosis in various cancers [36,37,38,39].

A transcription factor, *OCT4A* (*POU5F1*), an important regulator of pluripotency and stemness, has three known transcript variants, *OCT4A*, *OCT4B*, and *OCT4B1*, as well as various pseudogenes [40]. *OCT4A* expression has been reported in cancer stem cells, implicated in chemotherapy resistance, and linked to clinical outcomes in cancer patients [41,42,43,44].

*LIN28* (encoding two paralogs: LIN28A and LIN28B) plays a crucial role in the maintenance of pluripotency state and in the regulation of cell cycle, proliferation, tissue repair, microRNA biogenesis, and metabolism [45]. It has been demonstrated that an increased *LIN28* expression contributes to cancer development and progression, resistance to various anti-cancer therapies, and poor prognosis in numerous cancers [46].

A number of studies have shown elevated expression of miR-205-5p and *HMGA2* in EC [47,48,49,50,51,52]. Therefore, we also analyzed miR-205-5p and *HMGA2* expression levels to provide an additional validation of our measurements.

## 2. Materials and methods

### 2.1. Patients and Samples

**Figure 1:**
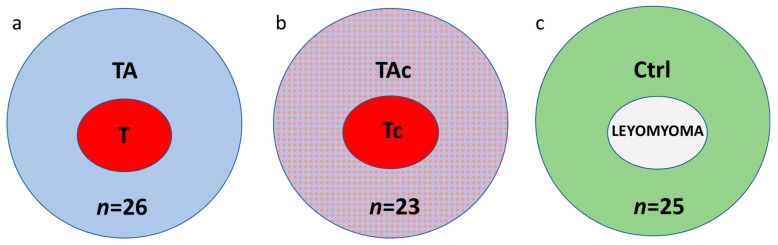
The studied samples from endometrial cancer (**a**,**b**) and cancer-free leiomyoma cases (**c**); a, paired tumor (T, red) and cancer cell-free tumor-adjacent samples (TA, blue); b, paired samples of tumors (Tc, red) and tumor-adjacent samples containing cancer cells (TAc, blue and red); c, control tissues, endometrial samples from cancer-free patients with leiomyomas (Control, Ctrl, green).

Paired samples of tumors and tumor-adjacent tissues were collected from 49 patients with histologically verified EC. Thirty-seven patients were diagnosed with endometrioid adenocarcinoma and 12 with other histological types (Table 1). In these series of paired samples, at histopathological examination of tumor adjacent tissues, 26 were found to be cancer-free (tumor–T and tumor-adjacent tissue–TA, respectively) (Figure 1a), while 23 contained cancer cells (tumor–Tc and tumor-adjacent tissue–TAc, respectively) (Figure 1b).

Twenty-five endometrial samples from cancer-free patients with leiomyomas (Control, Ctrl) were also collected (Figure 1c).

All samples of tumor-adjacent tissues were collected at least 1 cm from the tumor margins and snap-frozen. The patomorphological characteristics of the samples included the possible content of cancer cells and/or myometrium in the tumor-adjacent tissues from patients with EC.

Endometrial cancer cell lines AN-3-CA, MFE 280, and MFE 296 were obtained in 2011 from Deutsche Sammlung von Mikroorganismen und Zellkulturen (DSMZ, Braunschweig, Germany) and cell line HEC-1-A from American Type Culture Collection (ATCC, Manassas, VA, USA). Reauthentication of the cell lines was not necessary since these cell lines distributed by the repositories are subject to detailed characterization:

1/DSMZ: https://www.dsmz.de/collection/catalogue/human-and-animal-cell-lines (last accessed 12 August 2022)

2/ATCC: https://www.atcc.org/about-us/quality-commitment (last accessed 12 August 2022)

In addition, these cell lines were not passaged in our laboratory for more than six months after receipt before use in our study.

### 2.2. RNA Isolation, Reverse Transcription and Quantitative PCR

Total RNA was extracted using GeneMATRIX Universal RNA/miRNA RNA Purification Kit (EURx, Gdansk, Poland), following the manufacturer’s instructions. RNA integrity numbers (RINs) were checked with the use of 2100 Bioanalyzer (Agilent Technologies). RIN value of most samples exceeded 7 (Table S2). No differences in RNA quality were observed depending on sample source, i.e., T vs. TA and Tc vs. TAc.

The expression of mRNA transcripts and miR-205-5p was measured by the RT-qPCR method using 7900HT Fast Real-Time PCR System (Applied Biosystems, Carlsbad, CA 92008 USA) and 7500 Fast thermal cycler (Applied Biosystems Carlsbad, CA 92008 USA), respectively. The instruments and reagents were purchased from Applied Biosystems (Carlsbad, CA 92008 USA). TaqMan probe sets were used for *TWIST1* (Hs00361186_m1), *SNAI1* (Hs00195597_m1), *NR5A2* (Hs00187067_m1), *MYC* (Hs00905030_m1), *CXCR2* (Hs01011557_m1), *STK11* (Hs00975986_m1), *HMGA2* (Hs00171569_m1), and miR-205-5p (ID No. 000509) expression assessment. The other specific primers and TaqMan probe sets were designed using PrimerExpress software (Applied Biosystems, Carlsbad, CA 92008 USA) as follows: *LIN28A*, Forward 5′-TTCGGCTTCCTGTCCATGAC-3′, Reverse 5′-CCACTGCCTCACCCTCCTT-3′, Probe 6-FAM-5′-TTTGTGCACCAGAGTAA-3′-MGB; *POU5F1* (*OCT4*) isoforms, (a) *POU5F1* isoform A (OCT4A) Forward 5′-GGAGACCTCTCAGCCTGAGG-3′, Reverse 5′-TTGATGTCCTGGGACTCCTC-3′, Probe 6-FAM-5′-CAGGGGTGACGGTG-3′-MGB, (b) *POU5F1* isoform B (OCT4B) Forward 5′-AGACTATTCCTTGGGGCCAC-3′, Reverse 5′-GGCTGAATACCTTCCCAAATA-3′, Probe 6-FAM-5′-TGCCAAGCTCCTGAAGCA-3′-MGB, c) *POU5F1* isoform B1 (OCT4B1) Forward 5′-GTGCTCCCTCACTTTGCTTCTC-3′, Reverse 5′-TTTCTGCTTTGCATATCTCCTGAA-3′, Probe 6-FAM-5′-CAGGGAAGGTATTCAGCCA-3′-MGB. Based on the NormFinder_0953 algorithm results (Table S1), out of eight candidate genes, *PPIA* (Hs99999904_m1) and *RPLP0* (Hs99999902_m1) were selected as references for gene expression, while for miR-205-5p expression assessment *RNU44* (ID No. 001094) and *RNU48* (ID No. 001006) were used. The number of amplification cycles (CT value) for reference genes were similar between different series of samples.

### 2.3. Statistical Analysis

The relative expression levels were calculated using the ΔCt method. In statistical analyses, the Wilcoxon signed rank test was used for paired samples, and the Mann–Whitney rank sum test was used to compare groups of samples.

Statistical analyzes considering relationships between gene expression with clinico-pathological characteristics, i.e., stage, grade, histological type, relapse time, and 5-year survival, were carried out in the R environment (version: 3.6.1). The survival analysis was performed using the multivariate Cox proportional hazards models (the survival package for R, version: 3.2-11). All Cox models were also checked with respect to proportionality of hazards for each variable used. The prediction of treatment response in the experimental group of patients was analyzed by generating multivariate logistic regression models (R packages: stats (version: 3.6.1) and rms (version: 6.2-0)). In order to verify the discriminating capabilities of the Cox and logistic regression models, we performed their cross-validation in new data sets, generated from the original data by bootstrapping (with replacement) and subsequent comparison of areas under curves (AUCs) between the original and bootstrapped data sets, using the riskRegression package for R (version: 2020.12.8). All the analyses were performed not only in the entire group of tumors, but also in the subgroups with and without tumor cells in the tumor-adjacent tissue, and were adjusted for the clinical stage, histological grade, and type of tumor. Noteworthy, for all the analyzed genes, the expression was treated as a continuous variable to avoid arbitrary categorization of data, which could potentially lead to unreliable statistical results.

All *p*-values were considered significant at the statistical significance level (α) of 0.05.

## 3. Results

### 3.1. Gene Expression in Tumor-Adjacent Tissues and Tumors in Patients with EC and in Endometrial Tissue in Cancer-Free Patients with Leiomyoma

#### 3.1.1. *MYC*

**Figure 2:**
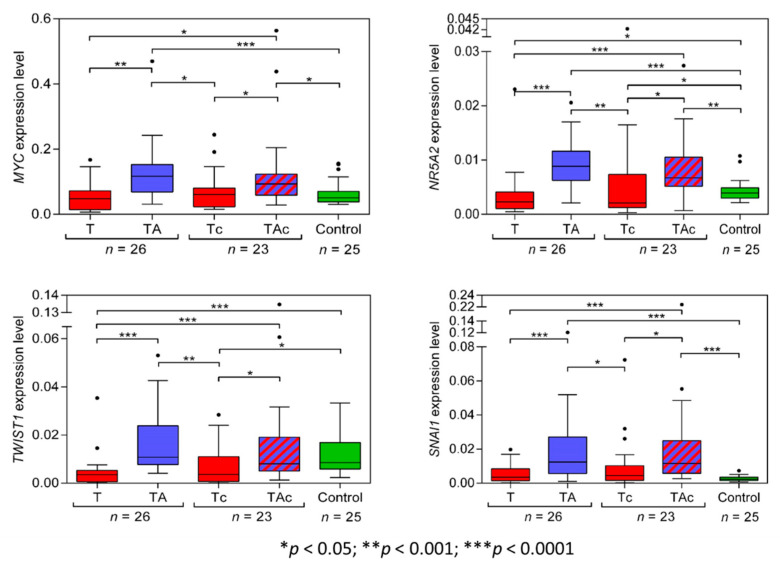
*MYC*, *NR5A2*, *TWIST1*, and *SNAI1* expression levels in tumors (T, red) and tumor-adjacent tissues without (TA, blue) or with (TAc, blue-dashed) cancer cell content in patients with EC, and in endometrial tissue in cancer-free patients with leiomyoma (Control, green).

The level of *MYC* expression was significantly higher in TA than in the matched T samples. *MYC* expression was similar in T, Tc, and Ctrl samples. *MYC* expression was also significantly higher in TAc than in the matched tumor samples and in TAc than in Ctrl samples (Figure 2, Table 2 and Supplementary Figure S1a).

#### 3.1.2. *NR5A2*

The expression level of *NR5A2* was significantly higher in TA than in the matched T samples, and in Ctrl than in T samples. It was also significantly higher in TAc than in the matched Tc samples, in TAc than in Ctrl samples, and in Ctrl than in Tc samples (Figure 2, Table 2 and Supplementary Figure S1b).

#### 3.1.3. *TWIST1*

The expression level of *TWIST1* in TA samples significantly exceeded that in the matched T samples and was also significantly higher in Ctrl than in T samples, but was similar in TA and in Ctrl samples. The expression level of *TWIST1* was also significantly higher in TAc samples than in the matched Tc samples and in Ctrl than in Tc samples, but it was similar in TAc and Ctrl samples (Figure 2, Table 2 and Supplementary Figure S1c).

#### 3.1.4. *SNAI1*

The expression level of *SNAI1* was significantly higher in TA than in the matched T, but did not significantly differ between T and Ctrl samples. The expression level of *SNAI1* was significantly higher in TAc than in the matched Tc samples and in Ctrl samples, but it was similar in Ctrl and Tc samples (Figure 2, Table 2 and Supplementary Figure S1d).

#### 3.1.5. *STK11(LKB1)*

**Figure 3:**
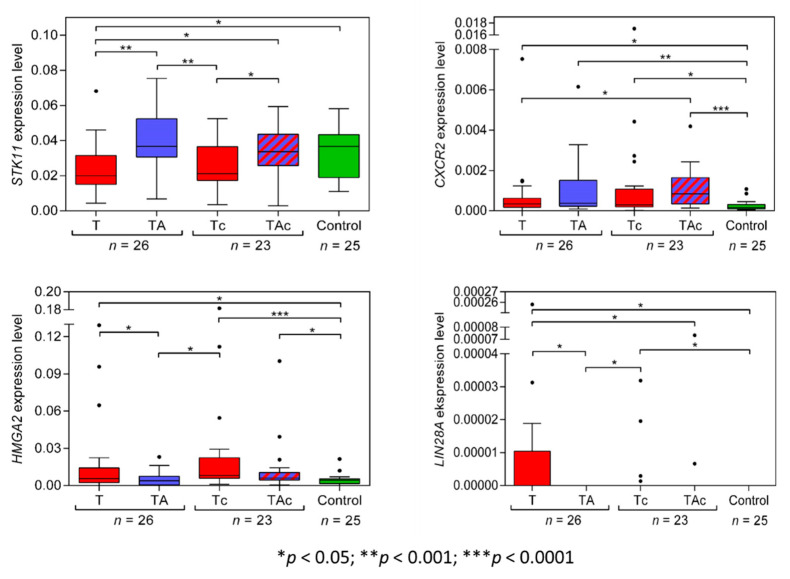
*STK11*, *CXCR2*, *HMGA2*, and *LIN28A* expression levels in tumors (T, red) and tumor-adjacent tissues without (TA, blue) or with (TAc, blue-dashed) cancer cell content in patients with EC, and in tumor-adjacent tissues in cancer-free patients with leiomyoma (Control, green).

*STK11*(*LKB1*) expression level was significantly higher in TA than in the matched T and in Ctrl than in T. *STK11*(*LKB1*) expression was similar in Ctrl vs. TA samples and vs. TAc samples. The expression level of *STK11*(*LKB1*) was significantly higher in TAc samples compared to the matched Tc samples, and in Ctrl it did not differ from that in Tc samples (Figure 3, Table 2 and Supplementary Figure S2a).

#### 3.1.6. *CXCR2*

The expression levels of *CXCR2* did not differ between T and the matched TA samples, but were significantly higher in T than in Ctrl samples. In TAc and in the matched Tc samples, no significant difference in the levels of *CXCR2* expression was found, but the levels were significantly higher in TAc and Tc samples than in Ctrl samples. (Figure 3, Table 2 and Supplementary Figure S2b).

#### 3.1.7. *HMGA2*

*HMGA2* expression levels were significantly higher in T than in the matched TA samples and in Ctrl samples. Similar *HMGA2* expression levels were found in TA and in Ctrl samples. Three T samples with the highest *HMGA2* expression derived from patients with: clear cell adenocarcinoma, endometrioid adenocarcinoma, and serous carcinoma. The level of *HMGA2* expression was similar in TAc and the matched T_C_ samples, but in TAc and in Tc samples it was significantly higher than in Ctrl samples (Figure 3, Table 2 and Supplementary Figure S2c).

#### 3.1.8. *LIN28A*

We found that only 9 out of 26 EC tumor samples and none of TA and Ctrl samples expressed *LIN28A.* Five of the *LIN28A*-positive tumor samples were endometrioid adenocarcinoma, and four were clear cell adenocarcinoma. *LIN28A* expression levels were significantly higher in T than in the matched TA samples (Figure 3, Table 2 and Supplementary Figure S2d).

#### 3.1.9. *POU5F1* Isoform *A (OCT4A)*

**Figure 4:**
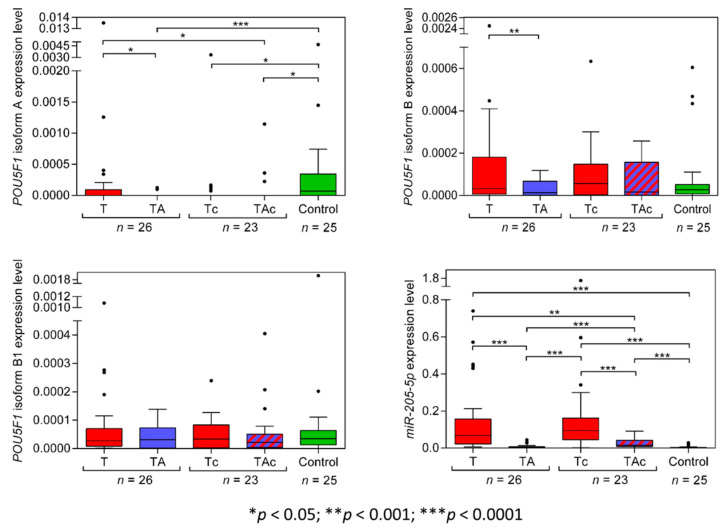
*POU5F1* (isoforms A, B, B1) and miR-205-5p expression levels in tumors (T, red) and tumor-adjacent tissues without (TA, blue) or with (TAc, blue-dashed) cancer cell content in patients with EC, and in endometrial tissue in cancer-free patients with leiomyoma (Control, green).

Among the matched T and TA samples from 26 EC patients, only 11 T and 2 TA samples expressed *POU5F1* isoform *A* (*OCT4A*), and the tumors derived from patients with: endometrioid adenocarcinoma (eight) and clear cell adenocarcinoma (three). Two *OCT4A*-positive TA samples derived from patients with endometrioid adenocarcinoma (Figure 4, Table 2, and Supplementary Figure S3a). *OCT4A* was expressed in 5 Tc samples only, diagnosed as clear cell adenocarcinoma (four) and carcinosarcoma (one). Three *OCT4A*-positive TAc samples derived from patients with endometrioid adenocarcinoma (two) and carcinosarcoma (one). A total of 14 out of 25 Ctrl samples expressed *OCT4A*. *OCT4A* expression levels were significantly higher in Ctrl than in TA samples. (Figure 4, Table 2 and Supplementary Figure S3a).

#### 3.1.10. *POU5F1* isoform *B (OCT4B)*

*POU5F1* isoform *B (OCT4B)* expression level was significantly lower in TA than in T samples. *OCT4B* expression did not differ between T and in Ctrl samples and between TA and Ctrl samples. (Figure 4, Table 2). The expression level of *OCT4B* was similar in TAc and the matched Tc samples, as well as in TAc vs. Ctrl samples, and in Tc vs. Ctrl samples (Figure 4, Table 2 and Supplementary Figure S3b).

#### 3.1.11. *POU5F1* isoform *B1 (OCT4B1)*

*OCT4B1* expression level was similar in all types of the analyzed samples, T vs. TA, T vs. Ctrl, TA vs. Ctrl, TAc vs. the matched Tc, TAc vs. Ctrl and Tc vs. Ctrl (Figure 4, Table 2 and Supplementary Figure S3c).

#### 3.1.12. miR-205-5p

The level of miR-205-5p expression was significantly higher in T than in the matched TA samples and was significantly higher in T than in Ctrl samples. miR-205-5p expression did not differ between TA samples and Ctrl samples (Figure 4, Table 2, and Supplementary Figure S3d). The expression level miR-205-5p was significantly higher in Tc samples than in the matched TAc samples, and in Ctrl samples, as well as in Tc than in Ctrl samples, and in TAc than in Ctrl samples. miR-205-5p expression was significantly higher in TAc compared to TA samples (Figure 4, Table 2 and Supplementary Figure S3d).

### 3.2. Gene Expression in Relation to Histological Results in Tumor-Adjacent Tissues

At histopathological examination, part of tumor-adjacent tissue samples from EC patients were found to contain various proportions of cancer and/or myometrial cells.

In 23 out of 49 EC patients, tumor-adjacent tissues contained cancer cells. There were no significant differences in the levels of *TWIST1*, *SNAI1*, *NR5A2*, *MYC*, *CXCR2*, *STK11*, *POU5F* isoforms *A*, *B*, *B1* (*OCT4A*, *OCT4B*, *OCT4B1*, respectively), and *HMGA2* in tumor-adjacent tissues, depending on cancer cell presence (TA vs. TAc), except miR-205-5p the expression of which was significantly higher in cancer cell-containing tumor-adjacent samples (Figure 2, Figure 3 and Figure 4). In tumor samples, none of the analyzed transcripts was differentially expressed depending on cancer cell presence in paired tumor-adjacent tissues (T vs. Tc) (Figure 2, Figure 3 and Figure 4).

In cancer cell-free EC-adjacent tissues (TA), the expression of *MYC*, *NR5A2*, *SNAI1*, and *CXCR2* was higher than in Ctrl samples (Table 2).

Out of 26 cancer cell-free TA samples, 10 contained 0–45% myometrial cells (TA-low-M), while the others contained at least 50% (TA-high-M). The levels of *CXCR2*, *MYC*, *NR5A2*, *POU5F* isoforms *B*, *B1* (*OCT4B*, *OCT4B1*), and miR-205-5p expression were similar in TA-high-M vs TA-low-M, while the levels of *HMGA2* and *SNAI1* expression was significantly higher and the level of *STK11* and *TWIST1* expression significantly lower in TA-low-M than in TA-high-M samples (Supplementary Figures S4–S6).

### 3.3. Relationships of Gene Expression with Clinical Data

We assessed relationships between the expression of the studied genes with stage, grade, histological type, relapse time, and 5-year survival in patients with EC. The only relationships revealed were as follows.

In patients with cancer-free tumor-adjacent tissue, higher *MYC* expression in the tumor increased the probability that tumor type was not endometrioid adenocarcinoma (OR 5.079, 95% CI [1.274–20.244], *p* = 0.021).

Higher TAc *NR5A2* expression in patients with cancer cells infiltrating the tumor-adjacent tissue increased the risk of death (HR 50.0557, 95% CI [2.105–1190.448], *p* = 0.016).

Higher miR-205-5p expression in tumor-adjacent tissue related to tumor cell presence in the tumor-adjacent tissue (univariate test: OR 2.338, 95% CI [1.202–4.547], *p* = 0.0124; multivariate test: OR 2.172, 95% CI [1.131–4.17], *p* = 0.020).

Higher *CXCR2* expression in tumor samples reduced the risk of recurrence in both the TA and TAc groups (univariate test: OR 0.0176, 95% CI [0.001–0.456], *p* = 0.015; multivariate test: OR 0.003, [0–0.508], *p* = 0.026).

### 3.4. Expression Levels of the Analyzed Transcripts in EC Cell Lines

The expression of *TWIST1*, *SNAI1*, *NR5A2*, *MYC*, *CXCR2*, *STK11*, *POU5F1* (*OCT4*, isoforms A, B, and B1), and *HMGA2* was studied in endometrial cancer cell lines, AN3 CA, HEC-1-A, MFE 280, and MFE 296. *HMGA2*, *MYC*, *STK11*, and *TWIST* expression was comparable to that in tumors from EC patients, while *CXCR2*, *NR5A2*, and *SNAI* expression was different from that in EC clinical samples. Inconsistent results on the expression of the other genes were observed (Supplementary Figures S1–S3).

## 4. Discussion

In EC, the expression of cancer-promoting genes, *MYC*, *NR5A2*, *SNAI1*, and *TWIST1*, was found to be significantly higher in tumor-adjacent tissues than in the matched tumors. The expression of all those genes, except *TWIST1*, was higher in tumor-adjacent tissues than in tissues neighboring uterine leiomyoma in cancer-free patients. Tumor-adjacent tissues from patients with leiomyoma were studied as the most adequate available control, as uterine biopsies of endometrium/myometrium from healthy women were inaccessible. We also present diverse expression of the analyzed genes in EC cell lines, not always reflecting the results in clinical material.

Actively released cancer cell-derived extracellular vesicles containing various functional molecules can influence the phenotypes of adjacent normal cells and promote cancer progression [53]. As recently shown, prostate cancer-derived large extracellular vesicles (oncosomes) facilitated intercellular communication through inducing high MYC activity in stromal cells [54], and human medulloblastoma cells with *MYC* amplification could release extracellular vesicles carrying *MYC* sequences [55]. Thus, extracellular vesicle-mediated induction of *MYC* expression may have contributed to the high *MYC* expression in the analyzed samples of EC-adjacent tissues. Noteworthy, a recently proposed model of MYC-dependent signal transfer from breast cancer cells to the surrounding cancer-associated fibroblasts involves exosome-transported miR-105, which downregulates the expression of *MXI1*, an inhibitor of MYC activity, and results in an increased MYC activity [56].

Increasing evidence also points to the important role of *MYC* upregulation in cell selection, also known as cell competition, leading to elimination of less competent cells, e.g., during embryonal development [57]. A possible role of cell competition has been suggested in field cancerization, a phenomenon characterized by phenotypic and genetic changes in tumor neighboring cells [57,58], and *MYC*-mediated “super competition” has been implicated in tumor progression [59]. Therefore, cell competition may contribute to the expansion of *MYC*-expressing cells in EC-adjacent tissues. We found that high expression of *MYC* was associated with more aggressive, non-endometrioid histology, as earlier suggested by Raeder et al. [60].

We showed significantly higher levels of *NR5A2* (also known as *LRH-1*) in EC-adjacent tissues than in paired tumors and in endometrial samples from cancer-free leiomyoma patients. We are the first to show *NR5A2* expression in clinical samples of EC. *NR5A2* expression in TA and TAc exceeded that in tumors, and in TAc, higher *NR5A2* expression increased the risk of death. In breast cancer, an increased *NR5A2* expression has been reported in tumor-adjacent adipose tissue as compared to normal breast tissue both from cancer-free patients and from breast cancer patients [61]. *NR5A2* is an estrogen receptor target gene [62], and NR5A2 is a key regulator of aromatase expression in breast cancer-associated adipose stromal fibroblasts [63]. Aromatase, a protein encoded by *CYP19A1* gene, is a key enzyme responsible for estrogen biosynthesis [64]. Noteworthy, an aromatase mRNA expression and activity has been found in EC-adjacent endometrium [65]. Thus, it is possible that increased *NR5A2* expression in EC-adjacent samples contributed to the aberrant aromatase expression/activity in peritumoral tissues, resulting in local estrogen production and EC progression. It has also been demonstrated that *NR5A2* may positively regulate *MYC* expression [66], therefore, the increased *NR5A2* expression may also contribute to the above-described *MYC* upregulation in EC-adjacent tissues. Noteworthy, in leiomyomas, a decreased expression of *MYC* and *NR5A2* in tumors compared to the corresponding myometrium has also been observed [67].

Noteworthy, Aran et al. have recently characterized a set of genes overexpressed in tumor-adjacent tissues as compared to tumors in several cancer types, and those genes strongly associated with various signaling pathways, including EMT [68]. Our data showed a higher expression of both *SNAI1* and *TWIST1* in EC-adjacent tissues than in the matched tumor samples. Similarly, in breast cancer, high expression of *SNAI1* and/or *TWIST1* in tumor-adjacent tissues has been demonstrated [10,69]. Montserrat et al. also observed higher expression of SNAI1 and TWIST1 in EC tumors than in endometrial samples obtained at hysterectomy from patients with uterine leiomyomas or prolapse, but EC-adjacent tissues have not been analyzed [51].

We demonstrated that in EC, tumor *STK11* expression level was lower than in the matched TA and in Ctrl samples. Our data implicating a tumor suppressor role of *STK11* in EC are in agreement with previous studies involving cell lines and animal models, where *STK11* deficiency was strongly associated with highly invasive phenotype of endometrial carcinoma cells [70,71,72].

Our data demonstrating elevated expression of miR-205-5p and *HMGA2* in EC tumors are in line with previous results [49,51,73,74], and provide an additional validation of the measurements we performed here. Accordingly, higher miR-205-5p expression in tumor-adjacent tissue related to cancer cell presence in the tumor-adjacent tissue.

The higher *POU5F1* isoform *A* (*OCT4A*) expression in EC in tumors than in the matched tumor-adjacent samples is consistent with previous literature data demonstrating *OCT4A* enrichment in EC tumor-initiating/cancer stem cells [75]. Surprisingly, *OCT4A* expression in TA samples were significantly lower compared to Ctrl samples derived from leiomyoma patients. The underlying mechanism of the difference is not clear. It is worth noting that recent data on patients with the most common *MED12*mt fibroid subtype showed a set of tumor-promoting genes to be upregulated in myometrium adjacent to tumors compared to normal myometrium [76].

Similar expression levels of all of the assessed transcripts, except miR-205-5p, were shown in cancer cell-free and cancer cell-containing tumor-adjacent tissues from EC patients. In full-thickness biopsy samples of relatively thin endometrium of postmenopausal women, the underlying myometrial cells are often present [77]. Considering the content of myometrial cells in TA tissues, samples with high percentage of myometrial cells expressed lower levels of *HMGA2* and *SNAI1* and higher levels of *STK11* and *TWIST1*, while all the other transcripts were similarly expressed. Hence, the possible contribution of cancer or myometrial cells in TA in the expression of all or most of the analyzed transcripts, respectively, seems limited. Interestingly, regarding *CXCR2* that was expressed at similar levels in T, TA, and TAc, patients with higher *CXCR2* expression in tumor samples presented reduced risk of recurrence independent of cancer cell presence in tumor surrounding. Explaining this issue requires further research.

The molecular etiology of EC and the pathogenic role of the demonstrated here overexpression of *MYC*, *NR5A2*, *SNAI1*, and *TWIST1* in tumor-adjacent tissues is not clear. The expression of *MYC*, *NR5A2*, and *SNAI1* was found to be higher in EC-adjacent tissues than in samples from cancer-free patients, which shows that tumor-surrounding tissues presented truly elevated expression, not just higher expression than in a tumor because of a decreased tumor expression. The increased expression of cancer-promoting genes found in tumor-surrounding tissues may represent pre/pro-cancerous alterations associated with field cancerization [58]. The tumor-adjacent tissue alterations may reflect the response of extratumoral microenvironment to the tumor. Aran et al., based on datasets from the Genotype-Tissue Expression project and The Cancer Genome Atlas, in tumor-adjacent tissues identified a set of genes strongly associated with pro-inflammatory signaling pathways to be specifically overexpressed, as compared to tumors [68]. Trujillo et al., in a study on breast cancer, identified a small set of genes involved in extracellular matrix remodeling, wound healing, and fibrosis that were significantly overexpressed in patient-matched, tumor-adjacent histologically normal breast tissues located 1 cm from the margins of breast adenocarcinomas, as compared to those in tissues located 5 cm from the visible tumor margin and in breast tissues from cancer-free patients [69]. Troester et al., in another study on breast cancer, demonstrated activation of wound response signature in the histologically normal tissue adjacent to tumors [78]. A possible contribution of stroma has also been suggested in the field cancerization phenomenon [79]. An accumulating body of data shows that molecular alterations in tumor-surrounding tissues can be orchestrated by the tumor. A study by Chatterjee et al. in breast cancer showed that fibroblasts isolated from tumor-adjacent tissue were tumor-activated and suppressed the clonogenic activity of normal breast epithelial progenitor cells while promoting the growth of malignant human mammary cells [80]. Recently, Amirrad et al. suggested a possible contribution of tumor-derived exosomes to the upregulation of EGR-1 and FASN in tumor-adjacent prostatic tissues [81]. The important role of extracellular vesicles in dictating the phenotypes of tumor-surrounding cells through such a paracrine (secretory) influence has recently been demonstrated in other studies [53].

In summary, our data provide important insights into the biology of tumors and the surrounding tissues in EC and highlight the need to characterize tumor-adjacent tissues. We showed significant abnormalities in the expression of cancer-promoting genes in tissues proximal to endometrial cancer, with higher expression of *MYC*, *NR5A2*, *SNAI1*, and *TWIST1*, in tumor-adjacent tissues than in tumors, which suggests field cancerization effect. Whether a field cancerization initially defined as cancer-preceding may also be tumor-induced and how far the tumor microenvironment reaches out remain open questions. Cancer-adjacent tissue, instead of being regarded as representative of the molecularly normal tissue (even if histologically normal) should rather be considered a potential target of anticancer therapy.

We are currently further exploring the molecular changes in EC tumor-adjacent tissues.

## Figures and Tables

**Table 1:** Characteristics of endometrial cancer patients.

**Patients with Cancer Cell-Free Tumor-Adjacent Tissues (TA), *n* = 26**					
**No.**	**Patient No.**	**Age**	**Histologic Type**	**Grade**	Stage *
1	12	57	Endometrioid adenocarcinoma	3	IA
2	23	70	Endometrioid adenocarcinoma	2	IA
3	24	57	Endometrioid adenocarcinoma	2/1	IA
4	25	84	Endometrioid adenocarcinoma	1	IIIC
5	26	72	Endometrioid adenocarcinoma	3	IIIC
6	29	58	Clear cell adenocarcinoma	3	IB
7	32	47	Clear cell adenocarcinoma	2	IB
8	33	60	Endometrioid adenocarcinoma	1	IB
9	35	61	Endometrioid adenocarcinoma	1	IA
10	36	70	Clear cell adenocarcinoma	2	IIIC
11	41	70	Serous carcinoma	1	II
12	42	73	Endometrioid adenocarcinoma	1	IIA
13	44	67	Endometrioid adenocarcinoma	1	IA
14	52	66	Carcinosarcoma	3	IB
15	54	75	Endometrioid adenocarcinoma	2	IB
16	56	50	Endometrioid adenocarcinoma	1	IA
17	58	57	Endometrioid adenocarcinoma	2	II
18	59	73	Endometrioid adenocarcinoma	2	IB
19	60	69	Endometrioid adenocarcinoma	2	IA
20	61	57	Endometrioid adenocarcinoma	2	IA
21	62	68	Clear cell adenocarcinoma	-	IB
22	63	53	Endometrioid adenocarcinoma	2	IA
23	67	68	Endometrioid adenocarcinoma	3	IA
24	69	75	Endometrioid adenocarcinoma	1	II
25	71	53	Endometrioid adenocarcinoma	3	III
26	73	65	Endometrioid adenocarcinoma	1	IB
**Patients with tumor-adjacent samples containing cancer cells (TAc), *n* = 23**					
**No.**	**Patient no.**	**Age**	**Histologic type**	**Grade**	**Stage**
1	11	53	Endometrioid adenocarcinoma	1	IB
2	16	55	Endometrioid adenocarcinoma	1	IB
3	17	54	Endometrioid adenocarcinoma	2	IB
4	18	75	Carcinosarcoma	3	I
5	19	72	Carcinosarcoma	3	I
6	20	61	Carcinosarcoma	2/3	I
7	22	63	Endometrioid adenocarcinoma	1	III
8	27	50	Endometrioid adenocarcinoma	2	I
9	31	53	Endometrioid adenocarcinoma	2	IB
10	34	85	Endometrioid adenocarcinoma	1	II
11	38	52	Endometrioid adenocarcinoma	1	II
12	39	60	Endometrioid adenocarcinoma	1	IIA
13	40	74	Endometrioid adenocarcinoma	2	II
14	46	75	Endometrioid adenocarcinoma partim mucinosum	1	II
15	47	80	Endometrioid adenocarcinoma	1	II
16	48	61	Endometrioid adenocarcinoma	1	II
17	50	78	Endometrioid adenocarcinoma	1	IB
18	53	74	Endometrioid adenocarcinoma	2	IB
19	57	63	Endometrioid adenocarcinoma	1	IB
20	64	54	Endometrioid adenocarcinoma	2	IB
21	65	72	Carcinosarcoma	3	II
22	66	73	Endometrioid adenocarcinoma	2	IB
23	68	76	Endometrioid adenocarcinoma	2	IB

* FIGO (International Federation of Gynecology and Obstetrics) staging system.

**Table 2:** Gene expression in tumor-adjacent tissues (TA) and tumors in patients with EC (T and Ts) and in endometrial tissue (TA and TAc) in cancer-free patients with leiomyoma (Control).

***p*-Values (Green Marks Statistical Significance)**												
	***MYC***	***NR5A2***	***TWIST1***	***SNAI1***	***STK11***	***CXCR2***	***HMGA2***	***LIN28A***	***POU5F1* isoform *A***	***POU5F1* isoform *B***	***POU5F1* isoform *B1***	**miR-205-5p**
**T vs TA**	0.0002	<0.0001	<0.0001	<0.0001	0.0002	0.2079	0.0493	0.0039	0.0068	0.001	0.2914	<0.0001
**Tc vs TAc**	0.0031	0.0101	0.0011	0.0027	0.0196	0.2467	0.1186	>0.9999	0.6875	0.601	0.1207	<0.0001
**T vs Control**	0.2053	0.0058	<0.0001	0.1857	0.0234	0.016	0.0421	0.0017	0.2174	0.6774	0.8409	<0.0001
**TA vs Control**	0.0001	<0.0001	0.0947	<0.0001	0.1506	0.0002	0.5311	>0.9999	<0.0001	0.4174	0.6432	0.1921
**Tc vs Control**	0.9511	0.0382	0.0107	0.0948	0.0868	0.0078	<0.0001	0.0455	0.0132	0.4241	0.8659	<0.0001
**TAc vs Control**	0.0079	0.0002	0.6637	<0.0001	>0.9999	<0.0001	0.0053	0.2243	0.0038	0.9224	0.3426	<0.0001
**T vs Tc**	0.1913	0.684	0.4591	0.4804	0.6407	0.7283	0.1715	0.2039	0.1643	0.9921	0.8194	0.5314
**TA vs TAc**	0.2356	0.1652	0.0526	0.8973	0.1845	0.2689	0.0946	0.2151	0.3834	0.3658	0.7125	0.0001
**T vs TAc**	0.0015	<0.0001	0.0001	0.0001	0.0059	0.0132	0.8817	0.0361	0.047	0.4579	0.4176	0.0003
**TA vs Tc**	0.0022	0.0002	0.0003	0.0037	0.001	0.4681	0.0034	0.0418	0.1391	0.0902	0.6152	<0.0001
**Medians of 2^−ΔCt^ values**												
	***MYC***	***NR5A2***	***TWIST1***	***SNAI1***	***STK11***	***CXCR2***	***HMGA2***	***LIN28A***	***POU5F1*** **isoform *A***	***POU5F1*** **isoform *B***	***POU5F1*** **isoform *B1***	**miR-205-5p**
**T**	0.04787	0.0023	0.00357	0.0035	0.02007	0.00034	0.00566	0	0	3.26 × 10^−5^	2.73 × 10^−5^	0.06781
**TA**	0.11679	0.00888	0.01078	0.0124	0.03672	0.00037	0.00401	0	0	1.28 × 10^−5^	3.13 × 10^−5^	0.00269
**Tc**	0.06117	0.00213	0.00362	0.00444	0.02114	0.00029	0.00819	0	0	5.63 × 10^−5^	3.33 × 10^−5^	0.09382
**TAc**	0.09343	0.00675	0.00802	0.01158	0.03373	0.00084	0.00609	0	0	1.66 × 10^−5^	2.22 × 10^−5^	0.01471
**Control**	0.05087	0.00395	0.00853	0.00225	0.03665	0.00015	0.00414	0	7.10 × 10^−5^	2.67 × 10^−5^	3.44 × 10^−5^	0.00199

HEX: #92d14f; RGB: rgba(146, 209, 79, 255).

## Data Availability

Most of the data generated or analyzed during this study are included in this published article and its supplementary information files. Detailed data and information are available from the corresponding author on reasonable request.

## Supplementary Materials

The following are available online at https://www.mdpi.com/2073-4425/13/9/1611/s1, Figure S1: *MYC* (a), *NR5A2* (b), *TWIST1* (c), and *SNAI1* (d) expression levels in tumor-adjacent tissues (TA, blue and TAc, blue-and-red pattern), in tumors (T and Tc, red) in patients with EC and in tumor-adjacent tissues in cancer-free patients with leiomyoma (Control, green), Figure S2: *STK11* (a), *CXCR2* (b), *HMGA2* (c), and *LIN28A* (d) expression levels in tumor-adjacent tissues (TA, blue and TAc, blue-and-red pattern), in tumors (T and Tc, red) in patients with EC and in tumor-adjacent tissues in cancer-free patients with leiomyoma (Control, green), Figure S3: *POU5F1* (isoforms A, B, B1) (a–c) and miR-205-5p (d) expression levels in tumor-adjacent tissues (TA, blue, and TAc, blue-and-red pattern), in tumors (T and Tc, red) in patients with EC and in tumor-adjacent tissues in cancer-free patients with leiomyoma (Control, green), Figure S4: *MYC*, *NR5A2*, *TWIST1*, and *SNAI1* expression in relation to histological results in tumor-adjacent tissues. Red horizontal lines mark significant differences assessed by the Wilcoxon signed rank test for paired samples, black horizontal lines mark significant differences assessed by the Mann–Whitney rank sum test comparing groups, Figure S5: *STK11*, *CXCR2*, *HMGA2*, and *LIN28A* expression in relation to histological results in tumor-adjacent tissues. Red horizontal lines mark significant differences assessed by the Wilcoxon signed rank test for paired samples, black horizontal lines mark significant differences assessed by the Mann–Whitney rank sum test comparing groups, Figure S6: *POU5F* (*OCT4A*), POU5F isoform B (*OCT4B*) and *POU5F* isoform *B1* (*OCT4B1*), and miR-205p expression in relation to histological results in tumor-adjacent tissues. Red horizontal lines mark significant differences assessed by the Wilcoxon signed rank test for paired samples, black horizontal lines mark significant differences assessed by the Mann–Whitney rank sum test comparing groups, Table S1: Reference gene candidate expression stability, Table S2: RNA Integrity Number (RIN) values of the studied samples.
