# Supplementary material for: Endometrial Cancer-Adjacent Tissues Express Higher Levels of Cancer-Promoting Genes than the Matched Tumors
Source: Genes (Basel). 2022 Sep 8;13(9):1611. doi: 10.3390/genes13091611 (PMC9527013; doi:10.3390/genes13091611)
Supplement: Supplementary file 1 [file genes-13-01611-s001.zip › genes-1840930-supplementary/genes-1840930-supplementary-Table 2.pdf]

Supplementary Table 2. RNA Integrity Numbers (RIN) of the studied samples.

Samples from patients with cancer cell-free tumor-adjacent tissues (TA), n=26			
No.	***Patient no.***	**RIN**	
		**T**	**TA**
1	12	9.3	9.1
2	23	8.7	8.4
3	24	9.8	8.2
4	25	9.4	9.5
5	26	10	9.6
6	29	NA	8.7
7	32	6,0	7.2
8	33	9.8	10
9	35	10	9.7
10	36	6.4	7,0
11	41	7,0	6.8
12	42	9.8	8.9
13	44	10	9.8
14	52	5.1	8.6
15	54	9.7	9.6
16	56	9.8	9.8
17	58	9.7	9.5
18	59	8.9	9.7
19	60	9.9	9.7
20	61	9.7	9.6
21	62	8.7	7.6
22	63	10	9.8
23	67	9.5	9.5
24	69	9.8	9.8
25	71	10	9.8
26	73	9.6	9.9

Samples from patients with tumor-adjacent tissues containing cancer cells (TAc), n=23			
No.	***Patient no.***	**RIN**	
		**Tc**	**TAc**
1	11	NA	NA
2	16	9.5	NA
3	17	9.4	NA
4	18	8.7	7.9
5	19	8	8.7
6	20	8.5	8.7
7	22	7.9	8.7
8	27	5.9	9.1
9	31	8.6	NA
10	34	7.1	7.3
11	38	7.7	8.9
12	39	7.4	7,0
13	40	7.7	6.5
14	46	6.8	NA
15	47	8,0	7.5
16	48	9.3	NA
17	50	7,0	7.6
18	53	8.2	7.6
19	57	7.5	4.8
20	64	4.8	7.5
21	65	NA	7.1
22	66	7.7	7,0
23	68	9.1	6.6

Samples from cancer-free patients with leiomyomas (C, Control), n=25		
No.	***Patient no.***	**RIN**
1	1C	7.2
2	2C	6.5
3	3C	8.1
4	4C	10
5	5C	9.1
6	6C	9.4
7	7C	9.5
8	8C	9.5
9	9C	9.5
10	10C	9.8
11	11C	NA
12	12C	NA
13	13C	NA
14	14C	10
15	15C	10
16	16C	10
17	17C	10
18	18C	10
19	19C	10
20	20C	10
21	21C	9.9
22	22C	9.7
23	23C	9.6
24	24C	10
25	25C	10

NA, Not Assessed
