# Supplementary material for: Endometrial Cancer-Adjacent Tissues Express Higher Levels of Cancer-Promoting Genes than the Matched Tumors
Source: Genes (Basel). 2022 Sep 8;13(9):1611. doi: 10.3390/genes13091611 (PMC9527013; doi:10.3390/genes13091611)
Supplement: Supplementary file 1 [file genes-13-01611-s001.zip › Captures for Supplementary Figures.pdf]

Supplementary Fig. S1. *MYC* (a), *NR5A2* (b), *TWIST1* (c) and *SNAI1* (d) expression levels in tumor-adjacent tissues (TA, blue and TAc, blue-and-red pattern), in tumors (T and Tc, red) in patients with EC and in tumor-adjacent tissues in cancer-free patients with leiomyoma (Control, green).

Supplementary Fig. S2. *STK11* (a), *CXCR2* (b), *HMGA2* (c) and *LIN28A* (d) expression levels in tumor-adjacent tissues (TA, blue and TAc, blue-and-red pattern), in tumors (T and Tc, red) in patients with EC and in tumor-adjacent tissues in cancer-free patients with leiomyoma (Control, green).

Supplementary Fig. S3. *POU5F1* (isoforms A, B, B1) (a, b and c) and miR-205-5p (d) expression levels in tumor-adjacent tissues (TA, blue, and TAc, blue-and-red pattern), in tumors (T and Tc, red) in patients with EC and in tumor-adjacent tissues in cancer-free patients with leiomyoma (Control, green).

Supplementary Fig. S4. *MYC*, *NR5A2*, *TWIST1* and *SNAI1* expression in relation to histological results in tumor-adjacent tissues. Red horizontal lines mark significant differences assessed by the Wilcoxon signed rank test for paired samples, black horizontal lines mark significant differences assessed by the Mann-Whitney rank sum test comparing groups.

Supplementary Fig. S5. *STK11*, *CXCR2*, *HMGA2* and *LIN28A* expression in relation to histological results in tumor-adjacent tissues. Red horizontal lines mark significant differences assessed by the Wilcoxon signed rank test for paired samples, black horizontal lines mark significant differences assessed by the Mann-Whitney rank sum test comparing groups.

Supplementary Fig. S6. *POU5F* (*OCT4A*), POU5F isoform B (*OCT4B*) and POU5F isoform B1 (*OCT4B1*) and miR-205p expression in relation to histological results in tumor-adjacent tissues. Red horizontal lines mark significant differences assessed by the Wilcoxon signed rank test for paired samples, black horizontal lines mark significant differences assessed by the Mann-Whitney rank sum test comparing groups.
