# Supplementary figures and images for: Endometrial Cancer-Adjacent Tissues Express Higher Levels of Cancer-Promoting Genes than the Matched Tumors
Source: Genes (Basel). 2022 Sep 8;13(9):1611. doi: 10.3390/genes13091611 (PMC9527013; doi:10.3390/genes13091611)

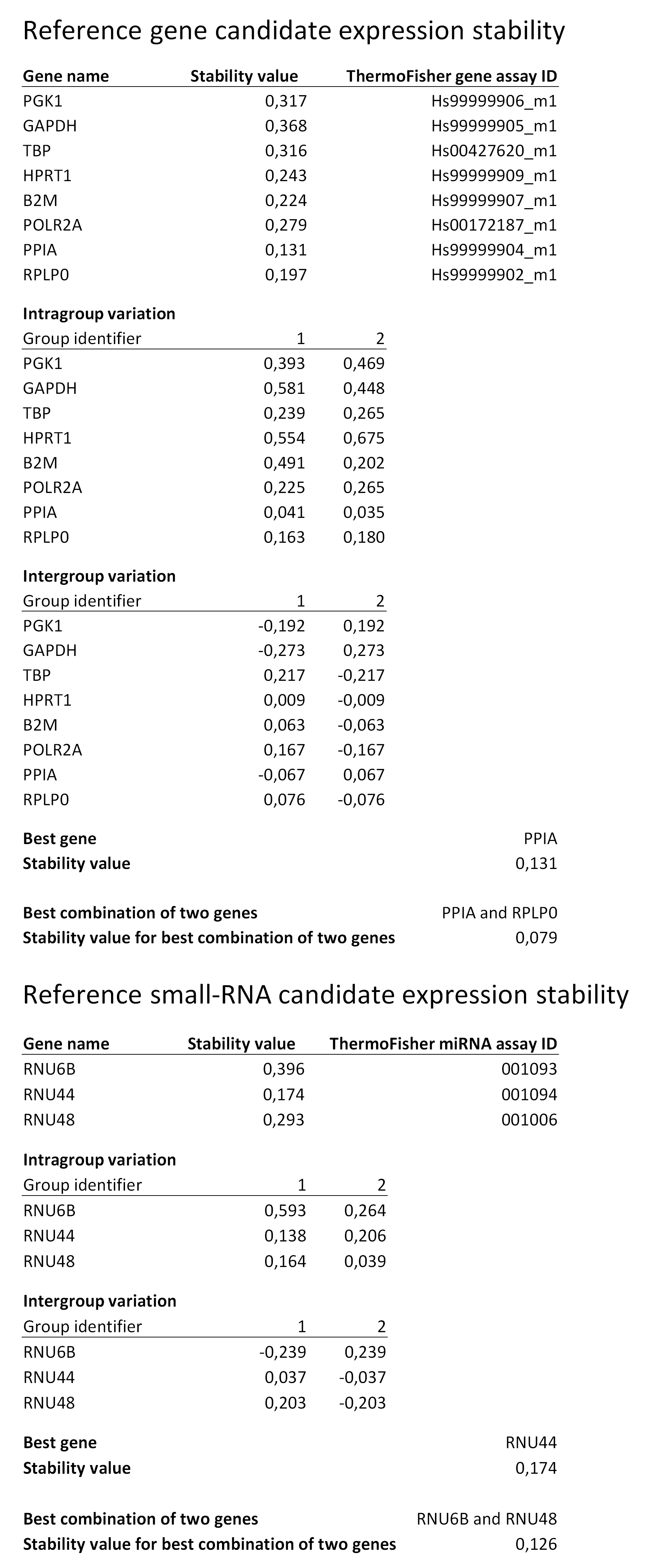

Supplement: Supplementary file 1 [file genes-13-01611-s001.zip › genes-1840930-supplementary/genes-1840930-supplementary - table1.jpg]

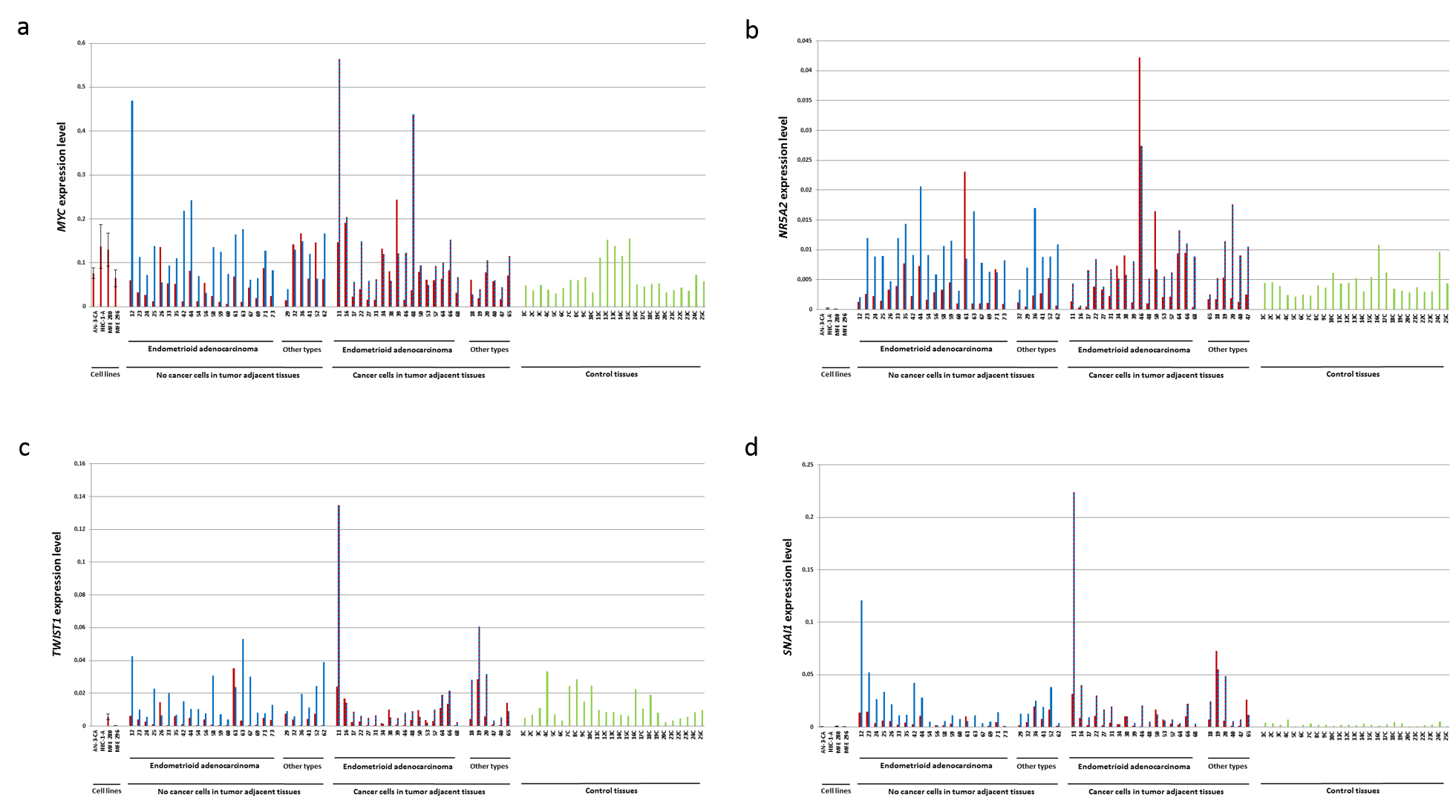

Supplement: Supplementary file 1 [file genes-13-01611-s001.zip › genes-1840930-supplementary/Supplementary Fig. S1.png]

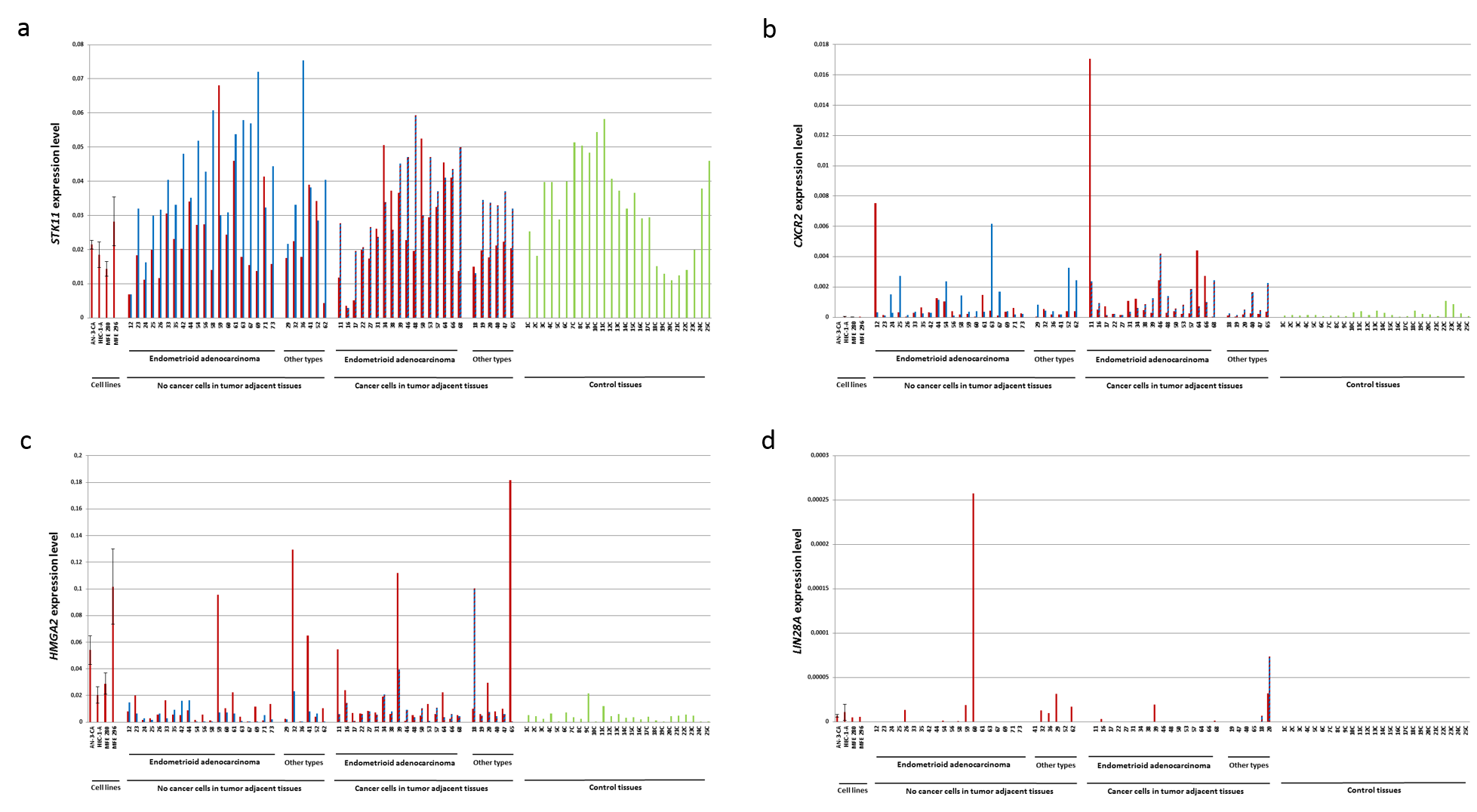

Supplement: Supplementary file 1 [file genes-13-01611-s001.zip › genes-1840930-supplementary/Supplementary Fig. S2.png]

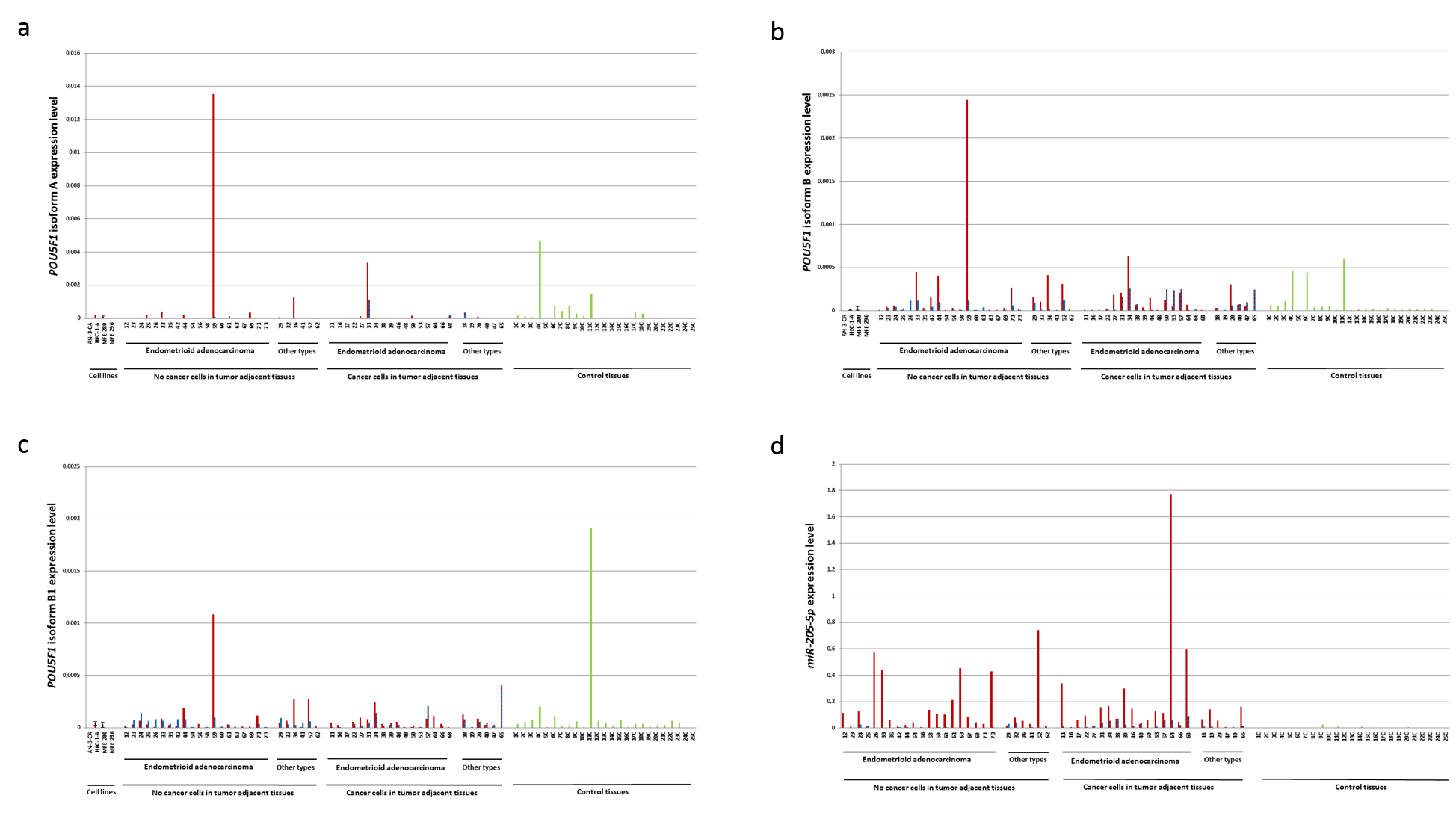

Supplement: Supplementary file 1 [file genes-13-01611-s001.zip › genes-1840930-supplementary/Supplementary Fig. S3.png]

## Slide 1
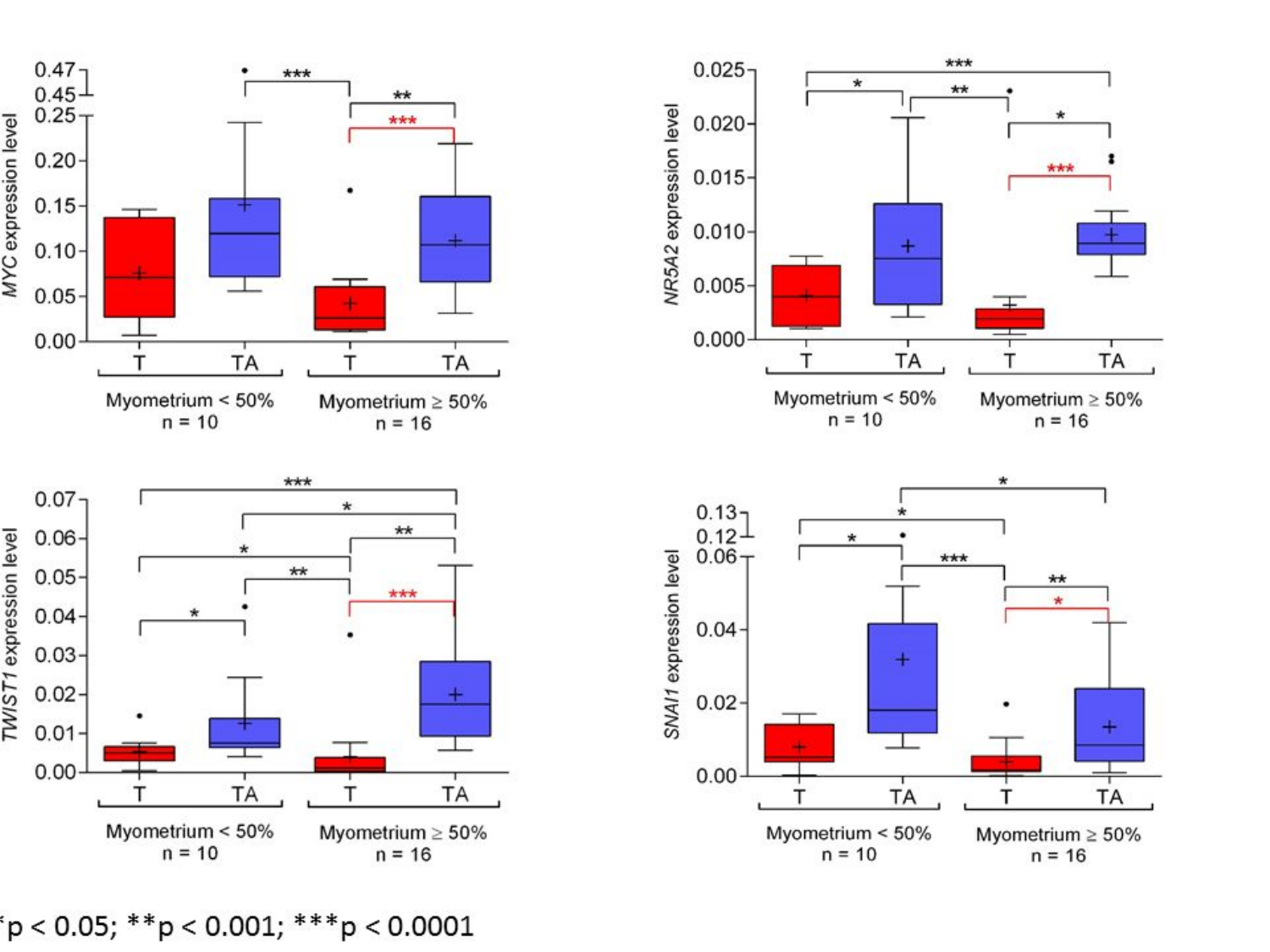

Supplement: Supplementary file 1 [file genes-13-01611-s001.zip › genes-1840930-supplementary/Supplementary Fig. S4.pptx]

## Slide 1
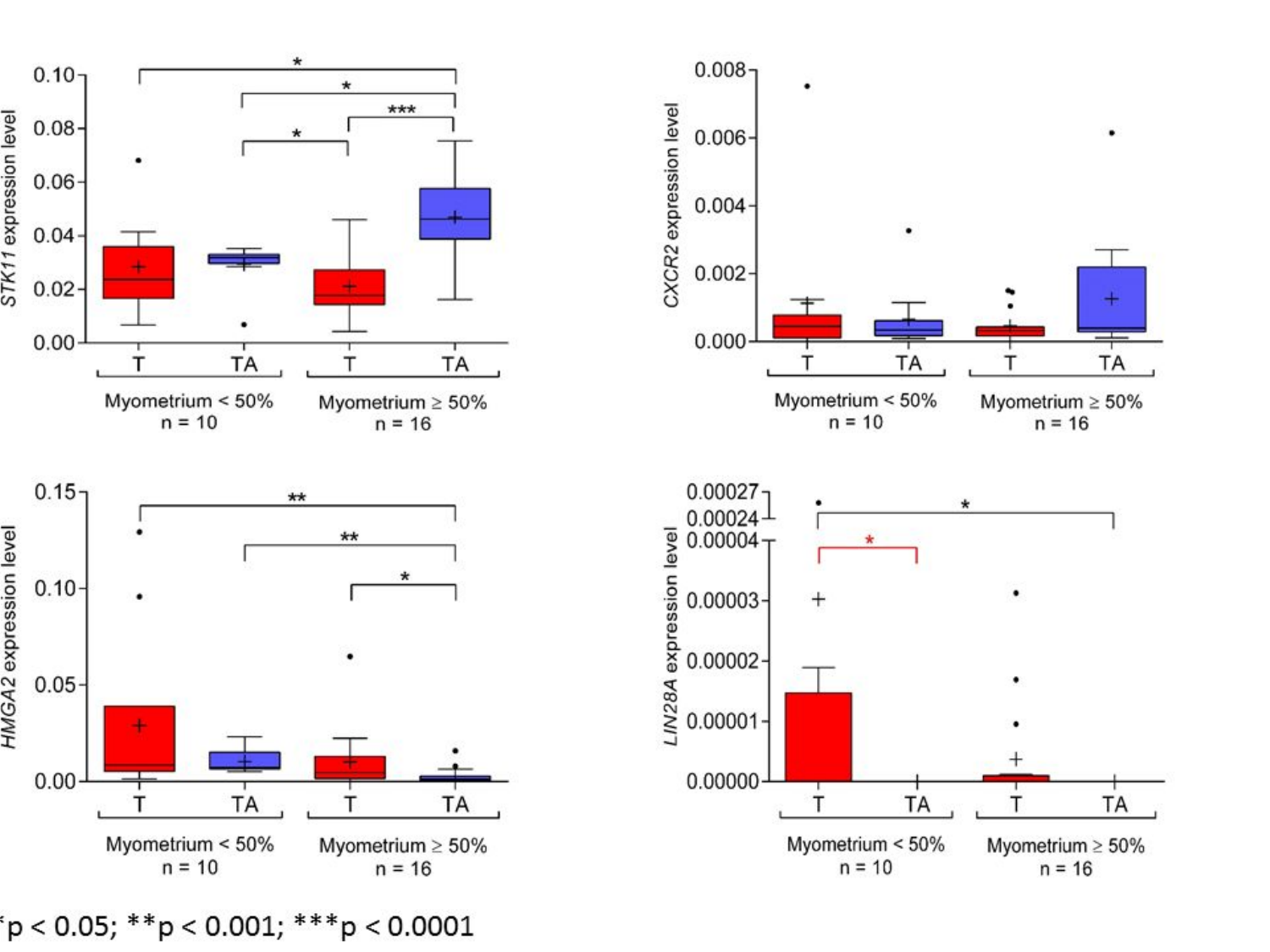

Supplement: Supplementary file 1 [file genes-13-01611-s001.zip › genes-1840930-supplementary/Supplementary Fig. S5.pptx]

## Slide 1
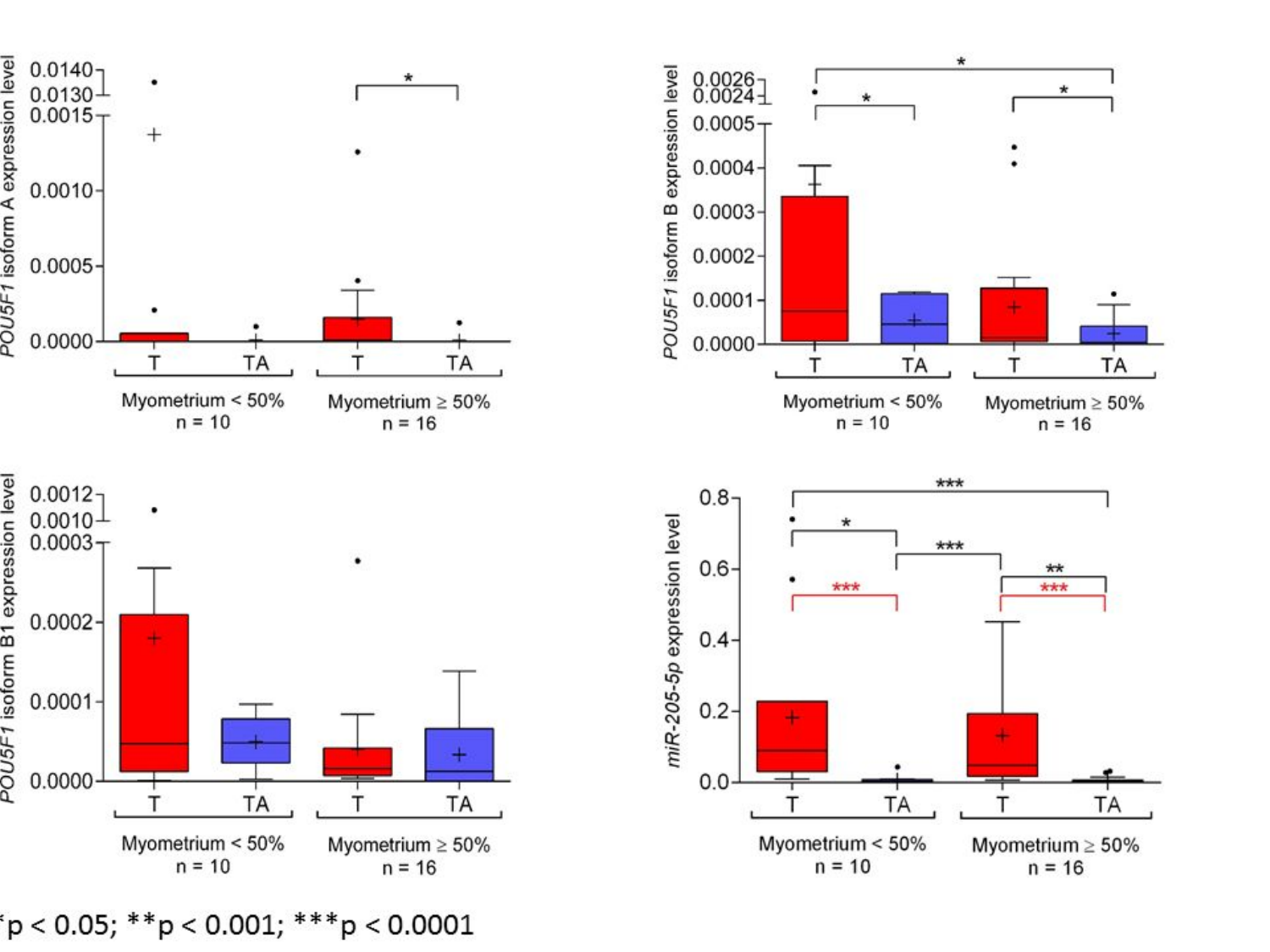

Supplement: Supplementary file 1 [file genes-13-01611-s001.zip › genes-1840930-supplementary/Supplementary Fig. S6.pptx]
